# Recent advances in addressing the market failure of new antimicrobials: Learnings from NICE's subscription-style payment model

**DOI:** 10.3389/fmedt.2023.1010247

**Published:** 2023-02-13

**Authors:** Marieke Schurer, Renu Patel, Marjolijn van Keep, Jake Horgan, Suzette Matthijsse, Matthew Madin-Warburton

**Affiliations:** ^1^ Lumanity, HEOR, Utrecht, the Netherlands; ^2^ Lumanity, HEOR, Sheffield, United Kingdom

**Keywords:** antimicrobial resistance, cost-Effectiveness, payment model, pull incentive, health technology assessment, reimbursement, cefiderocol, ceftazidime with avibactam

## Abstract

**Background:**

Antimicrobial resistance (AMR) is a growing threat to global health. With pathogenic bacteria inevitably becoming more resistant to existing antimicrobials, mortality and costs due to AMR will significantly increase over the next few decades if adequate action is not taken. A major challenge in addressing AMR is the lack of financial incentives for manufacturers to invest in developing new antimicrobials. This is partly because current approaches in health technology assessment (HTA) and standard modeling methods fail to capture the full value of antimicrobials.

**Aim:**

We explore recent reimbursement and payment frameworks, particularly pull incentives, aimed to address the market failures in antimicrobials. We focus on the “subscription-style” payment model recently used in the UK and discuss the learnings for other European countries.

**Methods:**

A pragmatic literature review was conducted to identify recent initiatives and frameworks between 2012 and 2021, across seven European markets. The National Institute for Health and Care Excellence (NICE) technology appraisals for cefiderocol and for ceftazidime with avibactam were reviewed to evaluate how the new UK model has been applied in practice and identify the key challenges.

**Conclusion:**

The UK and Sweden are the first European countries to pilot the feasibility of implementing pull incentives through fully and partially delinked payment models, respectively. The NICE appraisals highlighted the complexity and large areas of uncertainty of modeling antimicrobials. If HTA and value-based pricing are part of the future in tackling the market failure in AMR, European-level efforts may be needed to overcome some of the key challenges.

## 1. Introduction

Antimicrobial resistance (AMR) has become a major global issue (1, 2). While resistance is a natural biological phenomenon, it is accelerated by various factors, including poor infection control practices and global trade and travel. Overuse and misuse of antimicrobial medicines in humans and animals is perhaps the most exacerbating factor in this development (2). In 2019, an estimated 4.95 million people who died worldwide had at least one drug-resistant infection; 1.27 million of these deaths were directly attributable to AMR (3). Without adequate action, this is projected to rise to 10 million annual deaths by 2050 (4).

In 2015, the World Health Organization (WHO) adopted a global action plan for AMR using the One Health approach, which involves a collaborative effort between the human health, animal health, and agricultural sectors. This global action plan provides a framework for countries to address AMR, focusing on five key areas: (i) increasing awareness and understanding of AMR; (ii) increasing knowledge through surveillance and research; (iii) reducing the incidence of infection; (iv) optimizing the use of antimicrobial agents; and (v) ensuring sustainable investment in combating AMR through investing in new medicines, diagnostics, vaccines, and other interventions (5). Following on from this initiative, and after calls to action from the United Nations in 2016 (6) and 2021 (7), in March 2022 113 member states committed to tackling AMR (8). Most of these countries have developed their own national-level action plans, which are published online by the WHO (9).

Currently, member states are addressing AMR primarily by monitoring, limiting, and safeguarding the use of antimicrobials until strictly necessary, through surveillance and stewardship programs. Moreover, “push” incentives (financial incentives prior to regulatory approval) have been suggested and implemented to promote the development of new products—for example, by making research and development (R&D) funds available. Some initiatives include the Innovative Medicines Initiative's “New Drugs for Bad Bugs” (10), the AMR Action Fund (11), the Combating Antibiotic-Resistant Bacteria Biopharmaceutical Accelerator (CARB-X) (12), and the Global Antibiotic Research and Development Partnership (13, 14).

While these initiatives partially address the high R&D costs, they do not fully tackle the market failures post regulatory approval. A key challenge lies in the fact that the expected return on investment through current reimbursement methods is low. When a new antimicrobial becomes available, its use should be delayed for as long as possible to limit the development of AMR. Once resistance to existing drugs arises and the new antimicrobial is used, it will likely be joined by low-cost generics with the same mechanism of action, due to its patent expiring. Furthermore, there is uncertainty around how resistance—and thus the eligible population—will change over time, making it difficult for manufacturers to predict the long-term returns on a product (14, 15). As a consequence, the pipeline for antimicrobial agents remains insufficient (16), and several large pharmaceutical companies (Sanofi, Novartis, and AstraZeneca) have withdrawn from the antimicrobial market entirely (14, 15). There is therefore a critical need to ensure that investment into new antimicrobials is a commercially viable option for manufacturers, to provide clinicians with the necessary range of treatment options to tackle infections.

Recent studies have emphasized the need for “pull” incentives (financial incentives post regulatory approval) alongside push incentives to bridge the gap between high R&D costs and expected low returns on investment (15, 17–19)—for instance, through innovative payment models that partially or fully delink the payment of antimicrobial agents from their sales volume. In partially delinked payment models, manufacturers receive a financial reward when they successfully launch a new antimicrobial, in addition to their sales revenues. Fully delinked payment models provide manufacturers with an annual subscription fee based on the product's overall value, regardless of sales volume, for governments to have on-demand, unlimited access to the product (18). Several studies have investigated the required size of push and pull incentives to create an economically viable antimicrobial market, of which most suggested that a global figure of $1 billion would be required for pull incentives (18). A more recent study by Outterson concluded that this value may be even higher, with best estimates of $2.2 billion for partially delinked global market entry rewards and $4.2 billion for fully delinked global subscriptions over a 10-year period (18).

Health technology assessment (HTA) can play an important role in evaluating the value of new antimicrobial products and informing reimbursement and pricing decisions. However, this requires a shift in the commonly used concept of “value” in HTA in terms of cost per quality-adjusted life year (QALY) for the individual, to consider a broader definition of value and costs of novel antimicrobials (15, 20). The National Institute for Health and Care Excellence (NICE) in the UK, in collaboration with National Health Service (NHS) England and NHS Improvement, is investigating a new HTA process and a fully delinked payment model for antimicrobials (21). This new process has recently been tested in the evaluation of ceftazidime with avibactam (22) and cefiderocol (23). Similarly, the Public Health Agency of Sweden (PHAS) is conducting a pilot with a partially delinked payment model (24). Given the potential of such processes to set a precedent for future reimbursement and payment frameworks, an investigation of these early initiatives and their outcomes could highlight obstacles to implementing similar novel pricing mechanisms—and ways to avoid them.

In this review, we aimed to explore new initiatives and frameworks that have been used to tackle the market failures in AMR in Europe, with a focus on pull incentives and innovative payment models. We also reviewed the recent NICE appraisals of ceftazidime with avibactam and cefiderocol to (i) understand how a subscription-style payment model has been used in practice; (ii) identify the challenges posed by these analyses and how they had been addressed; and (iii) consider the learnings for other countries.

## 2. Materials and methods

A pragmatic literature review was conducted using PubMed® to identify innovative reimbursement and payment models, focusing on pull incentives that have either been implemented or are in the process of being implemented in Europe (see Supplementary Appendix A for the pragmatic search strategy) for antimicrobials. Electronic database searches were supplemented by searches on Google and websites of major HTA bodies in the EU4, the UK, the Netherlands, and Sweden. Searches were restricted to full-text articles published in English over the last 10 years.

In addition, publicly available documents from the NICE HTAs of cefiderocol and ceftazidime with avibactam were reviewed to understand the NICE Committee decisions and the key challenges identified in the appraisals.

## 3. Results

### 3.1. Recent initiatives addressing the market failure in new antimicrobials

Our pragmatic review did not identify any novel reimbursement or payment models for antimicrobials that are currently in use or being tested, apart from the subscription-style payment model recently implemented in England and the Swedish pilot of a partially delinked pull system. Gotham et al. (2021) (19) reported on reimbursement exceptions for antimicrobials in France and Germany. However, the French and German initiatives were excluded from this review, as they do not propose a separate evaluation process that formally relates to the reimbursement or payment of antimicrobials.

#### 3.1.1. Swedish supply-based procurement model

PHAS started a pilot scheme in 2018 to explore whether introducing a new, partially delinked, supply-based procurement model would be feasible to ensure access to existing and new antimicrobials. Manufacturers who participate in this agreement are rewarded with a minimum annual revenue on top of volume-based sales. In return, they are expected to guarantee supply of the product within pre-determined time frames. To meet this requirement, manufacturers must ensure that a certain volume of stock is available in Sweden. The availability-based annual revenue is set at SEK 4 million minus the annual sales. If the annual sales exceed SEK 4 million, manufacturers are still entitled to 10% of the minimum (SEK 4 million) annual revenue.

Five products are included in the pilot: Merck Sharp & Dohme's ceftolozane/tazobactam (Zerbaxa®) and imipenem/cilastatin/relebactam (Recarbrio®); Shionogi's cefiderocol (Fetcroja®); Menarini's meropenem/vaborbactam (Vaborem®); and InfectoPharm's fosfomycin. The products were selected for the pilot based on their risk of insufficient availability in Sweden and their special medical value. This includes products that have a good safety profile; that demonstrate good activity against critical pathogens included in the WHO's priority list from 2017 (25), such as carbapenem-resistant pathogens (*Acinetobacter baumannii*, *Pseudomonas aeruginosa*, and *Enterobacteriaceae*); and that can be used in at least two high-priority indications. Agreements lasted from 15 July 2020 to 15 July 2022, with a potential 2-year extension. The pilot will formally run until the end of 2022, after which it will be evaluated based on the availability of the selected products, the economic consequences of the pilot, and PHAS'experiences with the procurement process (24, 26).

#### 3.1.2. NHS England subscription-style payment model

NICE, in collaboration with NHS England and NHS Improvement, is testing a new HTA process and innovative payment model for antimicrobial products. Unlike the Swedish model, the UK model is demand based and fully delinks payment from sales volume. It does this by paying manufacturers a yearly fee to have on-demand access to their product, known as a subscription-based payment. The value of this fee is based on the product's value to the NHS, as assessed through an adapted HTA process.

NICE's pilot HTA process is guided by the Policy Research Unit in Economic Methods of Evaluation in Health & Social Care Interventions’ (EEPRU) framework for the evaluation of new antimicrobials (27, 28). The process considers not only the direct cost to the NHS of the antimicrobial product and the resulting QALY gains to the treated patient—outcomes commonly considered in HTA—but also five wider concepts of value beyond the treated patient. These are referred to as the STEDI values:

• **Spectrum value**: the value of replacing broad-spectrum antimicrobials with narrow-spectrum antimicrobials
• **Transmission value**: the ability to avoid onward spread of the pathogen in the population
• **Enablement value**: the value of enabling other treatments and procedures to take place, such as chemotherapy, organ transplant, and surgical procedures
• **Diversity value**: the value in having a range of treatment options available
• **Insurance value**: the value in having effective antimicrobials available in the event of a sudden increase of infections with pathogens resistant to existing antimicrobials

As well as including a broader definition of value, the adapted HTA process for antimicrobials differs from NICE's standard process with respect to its purpose and key outcomes of interest. The standard process considers whether a product should be reimbursed or not based on whether it offers value for money, whereas the proposed new evaluation process for antimicrobials provides guidance on the value of the product and informs commercial discussions (i.e., its level of payment). Rather than the incremental cost per QALY (the incremental cost-effectiveness ratio, or ICER) per patient, the new process seeks to express the overall value of antimicrobials in QALYs for the population as a whole, or a range of QALYs, depending on the uncertainty (28). This is done using a two-step approach: patient-level QALY gains are first determined, and then these QALY gains are multiplied by the eligible population for treatment at a population level (22, 23).

NICE piloted this new evaluation and payment model between January and February 2022 during the assessment of ceftazidime with avibactam and cefiderocol. Both assessments aimed to estimate the incremental population-level net health benefits of the products against the current standard of care, as measured in QALYs gained for the expected eligible population in England. The overarching objective of these appraisals was to inform the value-based payment of both products. The economic analysis was led by an academic group; in this case, it was led by the EEPRU. The key challenges and learnings from this pilot are summarized below.

### 3.2. NICE's appraisals of cefiderocol and ceftazidime with avibactam

#### 3.2.1. Challenge 1: defining the decision problem, the population of interest and comparators

In both HTAs, the marketing authorization of the products was broader than the population included in the EEPRU's analysis, which focused on high-value clinical scenarios (HVCSs). These scenarios referred to treating only those patients who would benefit most from the drug, in terms of mortality and health-related quality of life (HRQL). While it was acknowledged that the products might be used in a wider population than those defined in the HVCSs, restrictions and pragmatic decisions were required due to time, resource and evidential constraints (29, 30).

##### 3.2.1.1 Population of interest

The EEPRU investigated two different settings in the HVCSs of their patient-level analysis:

• **The microbiology-directed setting**, where the pathogen has been formally identified and its susceptibility tested and confirmed before treatment. This is applicable to non-critical infections only
• **The empiric setting**, in which treatment is urgently needed and initiated based on clinician suspicion of the pathogen and its mechanism of resistance

To inform the patient-level QALY gains, both HTAs considered infections with hospital-acquired and ventilator-associated pneumonia in the empiric setting (22, 23). While bloodstream infections were also considered frequent and clinically urgent infections, these were omitted from the analysis due to time constraints and because it is difficult to reliably distinguish between primary and secondary infections (31). The microbiology-directed setting also included complicated urinary tract infections (22, 23), as these have a slower clinical course (i.e., there is usually time to wait for test results before initiating treatment) and account for a large proportion of bloodstream infections (31).

Both appraisals investigated infections with carbapenem-resistant pathogens. In the ceftazidime with avibactam appraisal, the analysis was restricted to infections with *Enterobacterales* with OXA-48 mechanisms of resistance, while the cefiderocol appraisal was restricted to infections with *Enterobacterales* or *Pseudomonas aeruginosa* with metallo-*β*-lactamase mechanisms of resistance, given the limited available treatment options for these groups of patients.

In the population-level analysis of ceftazidime with avibactam and cefiderocol, the treatment-eligible population was expanded to include patients with bloodstream and intra-abdominal infections. In addition, a proportion of people with infections caused by *Stenotrophomonas maltophilia* were expected to be treated with cefiderocol*.* To account for this deviation between patient- and population-level analyses, the EEPRU made assumptions about the anticipated size of the population. Unsurprisingly, given the pragmatic methodological decisions made, the Committee concluded that the EEPRU's approximations of the current population size were underestimates.

##### 3.2.1.2. Comparators

Another issue in defining the decision problem was defining the appropriate comparators. This is particularly important, as the development of resistance over time is largely dependent on the treatments used. In both appraisals, a range of different comparators were considered potentially relevant, depending on the infection site, pathogen, mechanism of resistance and whether the treatment is used in the microbiology-directed or empiric setting (32). The final list of comparators is shown in Table 1. As outlined in the assessment reports of cefiderocol and ceftazidime with avibactam, the paucity of literature and reliance on *in vitro* data (see also Challenge 4) resulted in a simplified approach to modeling the comparators.

**Table 1:** Comparators in the HTAs of cefiderocol, and ceftazidime with avibactam.

Appraisal	Pathogen	Comparators: microbiology-directed setting	Comparators: empiric setting
Cefiderocol	*Enterobacterales*	• Tigecycline + colistin • Fosfomycin + colistin • Aztreonam + colistin • Aminoglycosides (gentamicin, amikacin, tobramycin)	• Tigecycline + colistin • Fosfomycin + colistin • Aztreonam + colistin • Aminoglycosides (gentamicin, amikacin, tobramycin)
	*Pseudomonas aeruginosa*	• Fosfomycin + colistin • Fosfomycin + meropenem	• Fosfomycin + colistin • Fosfomycin + meropenem
Ceftazidime with avibactam	*Enterobacterales*	• Meropenem + colistin • Fluoroquinolones (levofloxacin, ciprofloxacin) + meropenem • Aminoglycosides (gentamicin, amikacin, tobramycin) If low risk of ESBL and AmpC *β*-lactamase: • Cephalosporins (ceftriaxone, cefepime, ceftazidime) • Aztreonam + fosfomycin • Aztreonam + colistin HAP/VAP: • Tigecycline + colistin • Tigecycline + meropenem + colistin • Aminoglycosides (gentamicin, amikacin, tobramycin) may be used in combination with fosfomycin instead of as monotherapy	• Meropenem + colistin • Fluoroquinolones (levofloxacin, ciprofloxacin) + meropenem • Aminoglycosides (gentamicin, amikacin, tobramycin) + fosfomycin • Tigecycline + colistin • Tigecycline + meropenem + colistin

**Key**: ESBL, extended-spectrum β-lactamase; HAP, hospital associated pneumonia; VAP, ventilator associated pneumonia.

**Reference**: Adapted from the Assessment Reports of cefiderocol and ceftazidime with avibactam (32, 33).

In the microbiology-directed setting, patients were expected to be treated with the intervention either (1) if susceptibility testing showed the pathogen exhibits resistance to multiple existing therapies (multi-drug resistance); or (2) if the pathogen is susceptible only to treatments that are associated with an increased risk of nephrotoxicity, specifically colistin or aminoglycoside-based therapies. Because the EEPRU assumed that susceptibility is the only predictor of effectiveness (i.e., that *in vitro* susceptibility predicts *in vivo* clinical outcomes), the different comparators were not modeled individually. Instead, they compared outcomes (e.g., safety profile, length of hospital stay, mortality) of patients under existing care to those of patients with the new products available. In the model, one of the key benefits of cefiderocol and ceftazidime with avibactam becoming available was a reduced risk of developing acute kidney injury and chronic kidney disease, plus the long-term outcomes associated with these. Combination therapies were not included due to a lack of available evidence.

Comparators in the empiric setting were slightly different. Because treatment in the empiric setting is initially based on the suspected (rather than confirmed) pathogen mechanism causing the infection, patients' treatment pathways can differ—and, ultimately, patients may either be correctly or incorrectly treated based on the clinician's suspicion of the pathogen. This means that some patients will stay on their treatment initiated in the empiric setting once susceptibility test results become available, while others will switch treatments. Again, the EEPRU assumed that all treatments in the empiric setting had the same effectiveness, while safety profiles might differ. Therefore, in the model, comparators were grouped as either colistin-/aminoglycoside-based or not colistin-/aminoglycoside-based.

**Key learnings:** Modeling wide-spectrum antibiotics is complex, in part due to the many potential indications and comparators to consider. Within the context of HTA decision-making, groups responsible for reviewing the clinical and economic evidence have limited time and budget available. In addition, evidence on the populations relevant to these appraisals was limited. This forces any evaluation to consider a narrower scope than may be relevant to the products, inherently leading to pragmatic decision-making and, therefore, uncertainty in the true value of evaluated products.

A more holistic view on the potential uses of antimicrobials, with less emphasis on capturing the intricacies of specific use cases, may help to reduce uncertainty on the population level, while accepting a greater degree of uncertainty at the micro level. Whether this trade-off would reduce the overall uncertainty in the decision problem is subject to further research.

#### 3.2.2. Challenge 2: evaluating health outcomes

Traditional HTAs typically quantify patient outcomes using QALYs, which represent a standardized approach to measuring both length of life and quality of life. Quality of life is usually derived from utility values: numerical values representing the preference of a particular individual or society for a certain health state, with 0 and 1 typically equivalent to death and perfect health, respectively (34). As the direct utility impacts of infections are expected to be only short term—and therefore not a key model driver—these were not modeled by the EEPRU. Instead, their model assumes that patients in the HVCSs have more comorbidities (as measured by the Charlson comorbidity index; CCI) than the general population, based on evidence for similar populations. The EEPRU used studies that report utilities by CCI score to account for the HRQL decrements patients experience due to these underlying comorbidities. This resulted in an overall weighted utility value of 0.66 for the full population, equating to a 0.90 multiplier when compared with the general population utility values (32, 33, 35). This multiplier was then applied to the age- and gender-adjusted EQ-5D HRQL weights of the general UK population. After estimating patient-level QALY gains associated with the use of ceftazidime with avibactam or cefiderocol, these estimates were then scaled up based on the estimated size of the eligible population to obtain population-level outcomes.

Within the draft guidance of both ceftazidime with avibactam and cefiderocol, this approach to estimating patient utilities was neither discussed nor critiqued. However, it does have several limitations, a clear one being the lack of direct utility data. The approach also requires a number of inherent assumptions that may not necessarily be reflected in practice. In particular, it assumes that the cohort of patients receiving treatment will remain similar, in terms of the incidence and prevalence of comorbidities over time. This assumption may not reflect reality—for example, resistance patterns could result in a change in the treated cohort.

**Key learnings:** Within the antimicrobial space, using QALYs as a primary outcome measure in economic modeling is challenging for a number of reasons. First, obtaining quality-of-life data (such as patient-reported EQ-5D responses) is difficult. Trial designs for antimicrobials do not lend themselves for the collection of these data, as patients are typically acutely unwell and experiencing other conditions at the time of infection; therefore, direct quality-of-life data are often scarce or absent entirely. Second, rather than looking at the QALYs of individual patients (as in traditional HTA), subscription-based reimbursement models of AMR scenarios require the consideration of QALYs at the population level. This requires an accurate reflection of the number and distribution of patients who are eligible for treatment (invoking Challenges 1 and 3). Finally, the population of individuals likely to require antimicrobial treatment is a heterogeneous one—not only in terms of patient characteristics such as age and extent of co-morbidities, but also in terms of expression of disease and benefits of treatment.

Incorporating health outcomes is a key pillar of HTA processes and an essential requirement for any reimbursement methodology seeking to determine the value of a product relative to the rest of the healthcare system. Given the lack of available data and wider population-level considerations, determining health outcomes in antimicrobials is uniquely challenging. While many of these challenges remain intractable, further study may lead to more reliable estimates. For example, it is unlikely that reliable quality-of-life data will ever be available from antimicrobial clinical trials. However, these data could be obtained from patients retrospectively following discharge, or vignette studies involving (recovered) patients and treating clinicians could be undertaken.

#### 3.2.3. Challenge 3: understanding the current and future size of the patient population eligible for treatment and the emergence of resistance over time

One of the key challenges in assessing antimicrobials is understanding the size of the treatment-eligible population. This depends on both i) the growing number of people with infections that are eligible for treatment with the new product; and ii) the emergence of resistance to the new intervention and comparators over time, which itself is a product of how both existing and new products are used in practice. In other words, a level of forecasting about the expected size of the population is required, and this is accompanied by substantial levels of uncertainty.

##### 3.2.3.1. Current size of the treatment-eligible population

The current size of the eligible population in both HTAs was based on microbiology test results from the UK Health Security Agency Second-Generation Surveillance System (UKHSA SGSS), which covers 98% of English hospital laboratories and provides information on the mechanism of resistance and susceptibility to antimicrobials. Key limitations to using these data include: the lack of information about the site of infection; the fact that the database does not cover all hospitals; the fact that information regarding whether a product has been used in clinical practice is omitted; and the fact that the data are retrospective, and therefore may not reflect the current reality.

##### 3.2.3.2. Expected growth of the treatment-eligible population

The expected growth of the treatment-eligible population was predicted in both appraisals based on historical data from Public Health England's Antimicrobial Resistance and Healthcare Associated Infections (AMRHAI) national reference laboratory. The growth data were subsequently extrapolated over the modeled 20-year time horizon using two different approaches: one that assumed persistent growth over time, and one that assumed initial growth followed by stabilization. In both HTAs, the Committee concluded that persistent growth is more plausible than stabilization.

##### 3.2.3.3. Resistance to the intervention and comparators over time

As usage of an antimicrobial product increases, so does the development of resistance to the product. Resistance to ceftazidime with avibactam and cefiderocol, and how this resistance might progress over time, was initially based on historic data from the European Antimicrobial Resistance Surveillance Network linking usage with resistance for other antimicrobials. However, as the EEPRU deemed the outcome of this prediction unrealistic (ceftazidime with avibactam: 0.03% resistance over 20 years; cefiderocol: 0.04–0.16%), alternative assumptions were explored in the base case analysis, ranging from 1% to 30%. Assuming good stewardship, and in discussions with the EEPRU alongside the company and clinical experts, the Committee concluded that a 5% increase in resistance to both products over 20 years was a reasonable assumption.

Resistance to existing antimicrobials affects the usage of new products and thus also plays a role in the emergence of resistance to the new product. It is therefore important to consider the expected growth in resistance to all comparators over time. Despite the likely increase of resistance to comparators (using the principle that increased usage of antimicrobials results in increased resistance), the EEPRU assumed that resistance to comparators would remain constant over time, as historic evidence for England did not provide sufficient evidence of any growth in resistance. The Committee noted that “it was important to account for the benefits of being prepared for a catastrophic emergence of widespread multi-drug-resistant infections” (22, 23). To reflect this, the EEPRU modeled multiple scenarios in which a new multi-drug-resistant pathogen emerges and cefiderocol or ceftazidime with avibactam are the only effective treatments. Their scenario estimates were based on suggestions from a Committee member with expertise in infectious diseases and outcomes presented for individual infection sites.

The Committee considered that resistance to comparators was likely to increase, but that the EEPRU's scenario analysis was highly uncertain. Important limitations include the lack of population-level incremental net health benefits covering all infection sites, and the fact that the pathogens included in the base case analysis were not modeled in the scenario analysis. The Committee concluded that the model underestimated the benefits of both therapies by not accounting for increased resistance to comparators.

**Key learnings:** Given the random and spontaneous nature of pathogen mutation, there is a substantial level of uncertainty around how resistance to antimicrobial agents evolves over time. While it is possible to estimate an “average” course of resistance emergence (the approach taken by the EEPRU), it is impossible to predict all potential patterns of resistance emergence, which may result in very different outcomes. It is important to recognize that the impact of this uncertainty is not linear and that potential errors in these estimates can have an extensive impact on the costs and benefits to society. In the most extreme scenarios, if a new antimicrobial is not used to treat anyone, governments using subscription-style payment models might pay millions per year for a treatment that remains on the shelf. On the other end of the spectrum, if there is an outbreak in resistance to existing treatments and the new antimicrobial becomes the only available effective treatment, it could, theoretically, save humanity. While these are extreme scenarios, they do emphasize the importance of understanding and quantifying the level of uncertainty and range of potential scenarios. This raises the question whether future HTAs for antimicrobials should consider a wider range of different scenarios, rather than searching for the average treatment-eligible population.

#### 3.2.4. Challenge 4: estimating relative effectiveness

Another key challenge in assessing antimicrobials is the difficulty in obtaining clinical trial data that can be used for economic evaluation. The randomized controlled trials (RCTs) that were available were associated with the following issues: (1) they were typically not in the same population as the one of interest (e.g. results not broken down by infection site, or not carbapenem-resistant); (2) clinical outcomes were not in the form required for modeling (e.g. no information on susceptibility and how clinical outcomes vary by susceptibility); (3) evidence was typically only available for the empiric setting; and (4) there was uncertainty about the representativeness of the “usual care” arm.

In both appraisals, rather than using direct evidence from patient outcomes, the EEPRU assessed relative clinical effectiveness using *in vitro* data. It did this by using laboratory tests of patient samples of the pathogen to assess its susceptibility to the antimicrobial treatment being reviewed. The results of two published studies were used to link mortality and length of hospital stay with pathogen susceptibility to treatment, which allowed clinical outcomes to be modeled empirically. To model outcomes in the microbiology-directed treatment setting, between five and seven experts were consulted to predict the relationship between susceptibility data and clinical outcomes (mortality, length of stay, type of ward). However, this method was criticized by the Committee as it did not consider other factors affecting treatment efficacy and outcomes, such as the resistance mechanism causing the infection and the site of tissue penetration. Nonetheless, clinical experts accepted these data in the absence of better estimates, and the Committee concluded that susceptibility was a reasonable proxy for clinical outcomes even though it introduced uncertainty into the model.

**Key learnings:** Identification of reliable clinical effectiveness data remains a fundamental challenge. To sufficiently report outcomes by site, susceptibility, and antimicrobial used, a very large RCT would be required. Despite this, there are potential avenues to generating more appropriate evidence. For example, products with demonstrated *in vitro* effectiveness could be piloted in test case hospitals, and outcomes could be compared with those from analogous hospitals without access to these products to generate evidence for wider reimbursement. While less reliable than clinical trial data, this form of prospective real-world evidence could provide an improved data source, albeit one that would require substantial planning and investment.

#### 3.2.5. Challenge 5: incorporating the STEDI values

One of the aims of the HTAs was to consider the broader, more dynamic population benefits of antimicrobials, using the STEDI values. The ways in which each value was included (or not) in the appraisals are described below.

Spectrum value was not included in either appraisal. Clinical advisors and other stakeholders did not consider spectrum value to be significant for either cefiderocol or ceftazidime with avibactam, as both were considered to have a broad spectrum of activity. Modeling of transmission value was also absent in the appraisals. Clinical advisors indicated that the direction of effect of the therapies on transmission was uncertain, but that the overall magnitude of effect was expected to be small. This is because introducing a new effective drug for the treatment of multi-drug-resistant infections has several opposing effects. The NICE appraisal documents explain that if a drug reduces time in hospital, then this is expected to reduce transmission. However, more effective treatments could also increase time spent in hospital, and therefore transmission, by reducing mortality. The true effect is ultimately unknown.

Enablement value was captured in some aspects of the evaluations. Improved treatment of pre- and post-operative infections was included in the EEPRU's HVCSs and expected usage projections, despite some uncertainty around whether the analyses captured the full benefits of treating pre-operative infections. For example, the evaluation captured the value of enabling care to other patients (by treating infections that would otherwise have required healthcare resource use). However, other aspects of enablement value were not captured. Both products under review are associated with reduced renal toxicity when compared with existing antimicrobials, yet the analyses did not consider the extent to which cefiderocol and ceftazidime with avibactam would free up resources by reducing the need for dialysis. Similarly, the value created by reducing lost time and resources caused by procedures being cancelled because of infection was not captured. There was also no consideration of how the availability of effective treatments for resistant infections could allow hospital wards to stay open in the face of outbreaks, as this was deemed to be an unlikely scenario. The Committee acknowledged the challenges in modeling enablement value, but felt that some important aspects were neglected; it therefore concluded that the EEPRU's model had not fully captured this value.

Diverse prescribing strategies—such as randomly allocating patients with similar clinical indications to different treatments—were not included in the EEPRU's quantitative assessments of population-level incremental net health effects. This is because clinical advisors indicated that in the HVCSs, diverse prescribing strategies were unlikely to be appropriate due to the lack of safe and effective alternative treatments. For diversity value, clinical experts suggested that the EEPRU's model underestimated the benefit of providing an alternative treatment option when there are supply issues with other antimicrobials. The experts explained that this would be particularly important when treating severe infections in intensive care units, because patients are also likely to have organ failure and few treatment options available.

Neither appraisal fully accounted for the insurance value of the therapies. The EEPRU did not model a scenario where the use of cefiderocol or ceftazidime with avibactam is completely held back to preserve its effectiveness. In this case, the EEPRU believed that the scenarios modeled in the HVCSs reflected this form of insurance value, as they involve heavily restricting usage to preserve long-term effectiveness. While the EEPRU did consider a scenario in which the products under review are the only effective treatments against a newly emerging drug-resistant pathogen, the Committee noted that the analyses were based on adopting a risk-neutral perspective. However, when estimating the insurance value of an antimicrobial, taking a risk-averse perspective (i.e., paying more for the product than its estimated value to avoid unwanted future events) is likely to be more appropriate.

Taken together, the NICE Committee identified that the model had not fully captured the STEDI values and, therefore, the potential benefits of ceftazidime with avibactam and cefiderocol.

**Key learnings:** Accurate incorporation of the STEDI values is a highly complex task, requiring a great deal of modeling effort and expertise as well as data that are unlikely to exist in full. Efforts could be made to quantify certain elements outside of individual assessments; for example, aspects of enablement and insurance value are likely to be applicable and similar across other antimicrobials. Research could be undertaken at a higher level, and in advance of future assessments, to allow for more realistic and consistent incorporation of these values. Nonetheless, incorporation of the STEDI values remains a daunting task, and it is questionable whether the effort required to model and quantify them accurately is worth it when a more pragmatic (and less resource-heavy) approach, such as the one used in Sweden, could suffice.

#### 3.2.6. NICE committee outcomes

Using the Committee's preferred assumptions, the outcomes of the economic analysis from the EEPRU resulted in an incremental net health benefit of 5,400 QALYs for cefiderocol and 3,700 QALYs for ceftazidime with avibactam, over a 20-year time horizon in England. However, as the models likely underestimated the size of the eligible population for treatment, omitted consideration of the development of resistance to comparators, and insufficiently captured all aspects of value, the Committee applied several multipliers. In the case of cefiderocol, the incremental QALYs were doubled to reflect the expected larger population size, and another 50% increase was applied to cover the wider value of the product, resulting in a total of 16,200 QALYs (or 970 QALYs per year over a 10-year contract period, assuming a minimum benefit of 60%). Similarly, for ceftazidime with avibactam, the QALY gains were doubled and increased by a further 20%, to account for the larger population size and value, respectively, resulting in a total of 8,800 QALYs (or 530 QALYs over a 10-year contract period, assuming a minimum benefit of 60%).

**Key learnings:** The NICE appraisals of ceftazidime with avibactam and cefiderocol demonstrated the challenges in capturing the full value of antimicrobials. Despite a collaboration between two leading academic institutions' best efforts to model the STEDI values, the outcomes of the economic model were deemed insufficient to inform decision-making because they were expected to substantially underestimate the incremental benefits of both products. Experts were consulted throughout the appraisals to address areas of uncertainty, although this was carried out in an unstructured way, and the final estimated total benefits relied heavily on Committee deliberation and assumptions. The Committee ultimately resorted to applying arbitrary multipliers, and pragmatic decision-making, rather than considering the outcomes of the modeling exercise. This raises the question of whether it is worthwhile to attempt to formally model the incremental health benefits of antimicrobials within the current framework.

## 4. Discussion

The new approach to HTA and the innovative payment model applied by NICE and NHS England show the ambition to address the issues associated with HTA processes when evaluating antimicrobials, which typically result in a low expected return on investment for manufacturers. By capturing the overall value to society of the antimicrobials, and by basing the fees paid to the manufacturer for the subscription to use the product on this overall value, manufacturers are guaranteed reimbursement for their product at an appropriate level—even if it is completely held back from use to preserve its effectiveness.

The recent NICE pilot demonstrated that economic modeling in AMR is extremely challenging. Despite the efforts of two highly regarded academic institutions, the modeling approach did not sufficiently capture the full product value that would be required for informed decision-making. There are numerous reasons for this, including the sheer technical effort and time required for such a complex modeling task, as well as fundamental limitations in the data available. A recurring theme throughout the appraisals of ceftazidime with avibactam and cefiderocol was uncertainty. Given (1) the lack of reliable data on the current use of the antimicrobials being reviewed and the eligible populations for these; (2) the complexity of prediction needed to estimate future eligible populations and expected resistance over time; and (3) the reliance on *in vitro* data to estimate effectiveness, the economic model necessarily relies heavily on assumptions and is thus susceptible to a high level of uncertainty and bias.

While economic models are always a simplification of reality and include a degree of uncertainty, the complexity and challenges associated with modeling antimicrobials result in uncertainty vastly greater than that typically seen in health economic modeling exercises. The potential consequences of this uncertainty are much greater in antimicrobial products and could have a considerable impact on the wider society. During the NICE Committee meeting of both HTAs, it became apparent that, due to the limitations of the economic model, the cost-effectiveness outcomes played a smaller part in the decision-making process than they normally would in other appraisals. A large part of the Committee's decision was based on consultation with clinical experts and deliberation, rather than evidence from health economic analyses. In addition, Committee members with expertise in infectious diseases were involved in informing several estimates of scenario analyses in the economic model, as well as the decision-making process, which can be seen as a limitation.

Future HTAs in antimicrobials should consider the role of structured expert elicitation, as is now suggested by NICE for all their technology appraisals (36), to obtain more robust estimates of key parameters of interest (e.g., the emergence of highly resistant pathogens and the number of people affected by this) and quantify the uncertainty surrounding them. In the evaluations discussed here, no explicit consideration was given as to which experts would be most suitable to address each of the questions raised during the process. For example, eliciting information from epidemiologists regarding transmission value may be more appropriate than obtaining judgements from clinicians. Formalizing the approach to expert elicitation would result in more robust estimates, albeit not data derived. Finally, further research on how society values extreme (but highly unlikely) scenarios and how this affects their willingness to pay of antimicrobial products could provide a stronger foundation to inform value-based reimbursement and pricing decisions in the context of high uncertainty.

The draft guidance published by NICE for the two respective antibiotic regimens suggest QALY gains of 970 and 530 QALYs per year for cefiderocol and ceftazidime with avibactam, respectively. In both cases, this includes the application of a seemingly arbitrary multiplier, substantially increasing the estimates from the economic model. If NHS England were to pay £20,000 per QALY gained—the lower bound of the range of willingness-to-pay thresholds considered by NICE—this would result in annual payments of £19.4 million for cefiderocol and £10.6 million for ceftazidime with avibactam. Interestingly, this comes close to the annual subscription fee determined by Rex and Outterson in 2020 (37). Based on the estimate that a global pull incentive of up to $4 billion would be required for significant antibiotic innovation, Rex and Outterson estimated that, assuming that all countries in the G20 pay their fair share, NHS England would need to pay an annual subscription fee of £10 million per year per antibiotic product (18, 37).

The key benefit of NICE's subscription-style payment model is that it allows for reimbursement on the basis of a product's value to the healthcare system rather than volume of sales. However, NICE's pilot highlighted the challenges in determining this value and thus the appropriate level of reimbursement to provide. As the final decision relied largely on Committee deliberation and clinical input rather than quantitative outcomes from the economic model, it should be considered whether a simpler and more pragmatic approach, such as the supply-based model in Sweden, is a more viable solution. The approach taken in Sweden appears to be simpler than NICE's approach. It does not apply conventional HTA methods, and neither comparative effectiveness nor cost-effectiveness are considered. Instead, a sum of money is provided for antimicrobial products that offer a high medical value for which the manufacturer can guarantee sufficient stock and supply of the product within pre-determined time frames. The Swedish pilot is still ongoing; once it is completed, it would be of interest to reflect on the outcomes and the type of challenges that were faced with this approach, and whether ultimately the more complex approach attempted by NICE was advantageous.

If value-based pricing through traditional HTA is preferred, consideration should be given as to how to improve the cost-effectiveness estimates—and thus the determination of a product's value to the healthcare system and society. Given the complexity of modeling, it may not be reasonable to expect *de novo* modeling to be undertaken for every antimicrobial product in every market. A more centralized approach to economic modeling could be more appropriate. For example, an international network such as EUnetHTA could create a single approved modeling framework that can be adapted for products (38). The EEPRU used a single model for two distinct clinical sites, suggesting that this model might be a useful starting point. This would allow for more time, and budget, to be allocated to developing a robust model, which could be used for multiple products and jurisdictions. By establishing a standard approach and associated set of assumptions, this would also reduce the complexity of national-level HTA review. While any model is likely to contain inaccuracies and biases, using a single model for all products would reduce or eliminate these biases when considering products relative to each other. A similar approach has been taken in other complex disease areas—such as diabetes, where a limited number of non-product-specific models were developed and deployed across the majority of products and HTA assessments throughout Europe (39, 40).

Even with a centralized model, decision-making could be undertaken by individual jurisdictions, who would independently appraise model outcomes adapted for their region. Alternatively, decision-making could be conducted at a higher, centralized level (e.g., European), giving access across numerous jurisdictions *via* a single evaluation (e.g., through Beneluxa and similar initiatives) (41). This centralized approach would also be more likely to provide incentives to manufacturers to invest in AMR; there would be more certainty in expected reimbursement from a larger, more valuable market compared with separately negotiating with numerous individual authorities. Ultimately, as the challenge of AMR extends well beyond individual country borders, it raises the question of whether addressing the market failure for antimicrobials should also be considered at an international rather than a country level.
