# Supplementary material for: Recent advances in addressing the market failure of new antimicrobials: Learnings from NICE's subscription-style payment model
Source: Front Med Technol. 2023 Feb 13;5:1010247. doi: 10.3389/fmedt.2023.1010247 (PMC9969890; doi:10.3389/fmedt.2023.1010247)
Supplement: Supplementary file 1 [file Datasheet1.pdf]

Appendix A: Pragmatic search strategy

Search last updated: 29/07/22		
**No.**	**Search term (PubMed)**	**Hits retrieved**
#1	((antimicrobial*[Title]) OR (anti-microbial*[Title])) OR (antibiotic*[Title])	182,158
#2	(((((((reimburs*[Title]) OR (pay[Title])) OR (economic evaluation*[Title])) OR (HTA[Title])) OR (health technology assessment*[Title])) OR (pricing[Title])) OR (market failure*[Title])) OR (health economic*[Title])	27,111
**#3**	**#1 AND #2**	**113**
**Restrict to English language, past 10 years and full text only**		**62**
